# ASLIF: Accurate Lane-Area Perception Through Adaptive Fusion of Structural Lines and Instance Segmentation

**DOI:** 10.3390/s26206638

**Published:** 2026-10-20

**Authors:** Peng Liu, Wenzheng Jia, Litao Zhu, Zichuan Hu, Wen Dai

**Affiliations:** 1 School of Resources and Environmental Engineering, Anhui University, Hefei 230601, China; x24301154@stu.ahu.edu.cn; 2 Chang Jiang WuHu Waterway Division, Wuhu 241000, China; cjwhhdc0553@163.com; 3 School of Computer and Artificial Intelligence, Nanjing University of Finance and Economics, Nanjing 210023, China; 4 School of Geographical Sciences, Nanjing University of Information Science and Technology, Nanjing 210044, China; ho803785@student.reading.ac.uk (Z.H.); wen.dai@nuist.edu.cn (W.D.); 5 Reading Academy, Nanjing University of Information Science and Technology, Nanjing 210044, China

**Keywords:** lane-area perception, lane instance segmentation, lane-line detection, adaptive prediction-level fusion, intelligent transportation systems

## Abstract

Lane-level road inventory aims to jointly extract direction-consistent lane areas and visible lane counts from street-view images. However, existing line-based methods do not directly recover complete lane regions, whereas area-based methods may include irrelevant pavement. To address these challenges, we propose adaptive structural line–instance fusion (ASLIF), a prediction-level framework that combines structural line predictions with instance-level area predictions. ASLIF converts O2SFormer line predictions into regional masks and fuses them with the continuous regional response produced by Mask R-CNN. An image-level fusion coefficient adjusts the relative contributions of the two branches according to scene complexity, estimated lane density, and the difference in branch confidence. Experiments on a test set from Nanjing, China, show that ASLIF achieves a precision of 0.92, a micro-F1 of 0.90, a mean-F1 of 0.90, and a mean-IoU of 0.82, with the highest precision among the evaluated baseline semantic-segmentation methods. These results indicate that ASLIF provides a precision-oriented operating point that suppresses false-positive lane regions while retaining substantial regional coverage.

## 1. Introduction

Lane-level road inventory, fine-scale road-network modeling, and road-asset updating require direction-consistent lane areas and visible lane counts [1,2]. Recovering these attributes from a single forward-facing street-view image is difficult because lane markings are elongated but visually weak and may be degraded by occlusion, shadows, illumination changes, pavement wear, and perspective convergence [3,4,5,6]. Intersections, side-road merges, and multi-lane corridors further complicate the association of visible boundaries with the lane regions they delimit.

Modern lane detectors represent lane structure through pixel segmentation, row-anchor classification, key-point association, parametric curves, or sequence generation [7,8,9,10,11]. These approaches improve boundary localization, longitudinal continuity, and computational efficiency. Their typical outputs, however, remain sampled points, thin masks, or curves rather than complete, direction-specific lane regions.

For lane-level road inventory, line positions alone are insufficient because the output must also delineate the regions between adjacent boundaries and identify the number of visible, direction-consistent lanes [1,2,12,13]. Line-based methods preserve longitudinal geometry, but converting lines into regions becomes unstable when boundaries are missing, incorrectly paired, or compressed by perspective [14,15,16,17]. Semantic segmentation produces continuous regions but merges adjacent lanes. In contrast, instance segmentation distinguishes individual lanes but is vulnerable to fragmented masks, duplicate instances, and false-positive responses from pavement, medians, and non-motorized lanes [12,13,18,19,20]. Joint boundary–region modeling demonstrates the complementarity of these two representations [13,17] but does not establish which should dominate under given imaging conditions. Roberts et al. [12] focus on lane-instance segmentation, whereas Zhang et al. [17] jointly model lane segmentation and lane-boundary detection within a unified network. Adaptive structural line–instance fusion (ASLIF) instead operates at the prediction level by mapping independently generated instance-level area and lane-line predictions into a common regional space and adjusting their relative contributions according to scene category, lane density, and branch-confidence difference.

The remaining challenge is to regulate the contributions of the two prediction types as their error patterns vary across images. Instance masks are often more informative when lane regions are clearly bounded, whereas line structure may remain useful under partial occlusion or a long-range perspective. Union, intersection, and fixed weighting cannot respond to these changes, while uncalibrated confidence scores are not necessarily comparable across independently trained branches [21,22,23]. An interpretable image-conditioned fusion rule may therefore provide a useful operating point, but its value should be established against single-branch, fixed-fusion, and cue-specific adaptive baselines.

To address these challenges, we develop adaptive structural line–instance fusion (ASLIF), a prediction-level framework that maps line-derived structural predictions and instance-level area predictions into a common regional-mask space. ASLIF uses scene category, estimated lane density, and branch-confidence difference to compute an image-level fusion coefficient.

The main contributions of this work are summarized as follows:

- ASLIF provides a prediction-level framework for the joint recovery of direction-consistent lane areas and visible lane counts, converting discrete O2SFormer line predictions into a regional representation compatible with Mask R-CNN instance masks.
- An interpretable, scene-conditioned fusion rule determines an image-level fusion coefficient from scene category, estimated lane density, and branch-confidence difference to combine structural and regional predictions.
- Experiments on a held-out study-area test set compare ASLIF with single-branch, logical-fusion, and single-cue alternatives and assess its two outputs separately. Pixel-level metrics and per-image analyses quantify the precision advantage and recall trade-off of the fused mask at the reported operating points, while lane-count error assesses the visible-lane count estimated before fusion.

The remainder of this paper is organized as follows. Section 2 reviews related work, Section 3 presents the proposed ASLIF framework, Section 4 describes the experimental setup and results, Section 5 discusses the findings and limitations, and Section 6 concludes the paper.

## 2. Related Work

### 2.1. Lane-Line Detection and Structured Representation

Lane-line detection has progressed from handcrafted color, gradient, edge, Hough-transform, and curve-fitting pipelines to learned segmentation, anchor, key-point, parametric-curve, and sequence representations [3,4,24]. Classical methods remain interpretable but are sensitive to shadows, worn markings, occlusion, and glare [4,25]. In contrast, learned methods improve longitudinal representation and robustness but still usually output points, curves, or thin masks rather than complete direction-consistent lane regions [6,9,10,11,15,26,27,28,29,30,31,32,33].

Deep learning has introduced several methodological approaches to lane-line detection [3,24]. Semantic segmentation methods such as SCNN and LaneNet reformulate lane detection as pixel-wise classification, capturing rich local details but often suffering from fragmented lane-line predictions and difficulty separating instances [7,18]. Row-anchor classification methods such as Ultra Fast Lane Detection predict lane points at predefined row positions, offering high inference efficiency and explicit structural constraints. However, they adapt less well to irregular road layouts and complex topology [9]. Anchor-based, line-proposal, and curve-parameter methods such as LaneATT and CLRNet produce compact geometric representations and enhance longitudinal continuity, but their ability to represent branches, occlusion, and irregular lane boundaries remains limited by the parameterization [11,15]. Key-point, conditional-prediction, Transformer, and sequence-generation methods, including CondLaneNet, LSTR, O2SFormer, and Lane2Seq, further strengthen global modeling, instance matching, and structural reasoning [10,31,32,33].

Although these approaches have advanced lane-boundary localization and modeling of longitudinal structure, their outputs are still usually discrete point sets, parametric curves, or thin-line masks [9,10,11,15,31,32,33]. Such representations describe lane position and direction, but they do not directly provide complete lane areas [12,13,17,18,19]. When lane lines are missed, occluded, or compressed by a long-range perspective, pairing adjacent boundaries becomes unstable, and line-to-area conversion can be incomplete or shifted [12,14,17]. Lane-line detection therefore provides important structural priors, but line-level prediction alone is insufficient for lane-area perception and lane-level structural analysis [17,32].

### 2.2. Lane-Area Segmentation and Instance-Level Extraction

Unlike lane-line detection, lane-area segmentation directly models the pixel coverage of road or lane regions. Semantic segmentation provides a principal approach to this task. Representative methods include FCN [34], SegNet [35], ENet [36], ERFNet [37], the DeepLab series [38,39], PIDNet [40], SETR [41], SegFormer [42], SegNeXt [43], and Mask2Former [44]. Encoder–decoder architectures recover spatially coherent regions [34,35,38,39]. Lightweight networks improve real-time performance [36,37,40]. Multi-scale context methods enhance semantic representation in complex scenes [38,39,42,43]. Transformer-based and newer segmentation architectures further improve global dependency modeling [41,42,44]. The main advantage of these methods is that they output continuous regional masks, which support lane-area coverage and completeness [34,35,38,39].

Semantic segmentation, however, usually merges multiple lanes of the same class into a single region. It cannot directly provide separate lane instances, lane counts, or instance-level geometry. When the task requires lane counting, adjacent-lane separation, or structured post-processing, semantic area prediction is limited by ambiguity in instance boundaries [34,35,38,39]. Instance segmentation methods such as Mask R-CNN can generate separate masks for different lanes and also provide bounding boxes, confidence scores, and instance counts. They are therefore better suited to lane-count estimation and instance-level structural analysis [19].

Lane areas are elongated, low-texture objects with strong perspective variation and incomplete boundaries. These characteristics create additional challenges for instance segmentation [12,13,17,18,19]. Lane masks may be truncated, fragmented, or locally missing. Adjacent lanes may merge or be detected repeatedly. Pavement texture, medians, and non-motorized lanes may also be misidentified as additional target lanes. In complex scenes, instance segmentation can over-predict, affecting lane counting and subsequent area fusion [12,18,19]. Semantic segmentation therefore provides strong area coverage but lacks instance-level lane structure, whereas instance segmentation separates lanes but is vulnerable to elongated-object morphology and local texture interference [19,34,44]. A single area-segmentation paradigm is thus unlikely to ensure area continuity, instance discrimination, and reliable lane counting simultaneously.

### 2.3. Scene-Aware Adaptive Multi-Model Fusion

Different models produce complementary outputs and exhibit distinct error patterns, making multi-model fusion an important approach to improving the stability of road understanding [21]. Existing fusion methods can be divided into data-level, feature-level, prediction-level, and decision-level fusion. This study focuses on prediction-level fusion, where predictions from different visual models are combined in a common output space to exploit the complementarity between line-level structural prediction and instance-level area prediction [21].

Common prediction-fusion strategies include union, intersection, fixed-weight averaging, confidence-based weighting, and learned gating. Mixture-of-experts models provide a direct precedent for input-conditioned gating [22]. Geometric joint lane segmentation and boundary-aware segmentation offer precedents for incorporating structural constraints [17,40]. Confidence-based weighting also requires careful consideration of calibration [23]. Together, these approaches motivate the interpretable, image-conditioned fusion rule used in this study. Learned or spatially varying gating remains an important alternative for future comparison.

In lane-area perception, scene conditions strongly affect model reliability. On ordinary roads with clear markings, instance segmentation often provides accurate area and count information. In scenes with occlusion, weak boundaries, or pronounced perspective effects, line-level detection is more useful for maintaining longitudinal structure. In multi-lane and complex urban roads, lane density affects the stability of both instance segmentation and line-level detection [5,6,12,14,31]. Even within the same scene category, model confidence, visible lane count, and local occlusion can vary substantially across images [21,23,45]. Fixed fusion strategies therefore cannot fully adapt to image-level road conditions [21,22,23,45].

Prediction-level fusion between line-derived structure and instance-level lane regions has received less direct attention than improvements to either branch alone [3,16]. Existing strategies include fixed weights, logical operations, confidence-based rules, and learned aggregation [21,22,23,45]. ASLIF examines an interpretable alternative by converting O2SFormer line predictions into regional masks and fusing them with Mask R-CNN instance masks using scene category, estimated lane density, and branch-confidence difference. The experiments therefore need to distinguish gains from the common regional representation, the three conditioning cues, and the final thresholding operation.

## 3. Methodology

### 3.1. Overview

**Figure 1:**
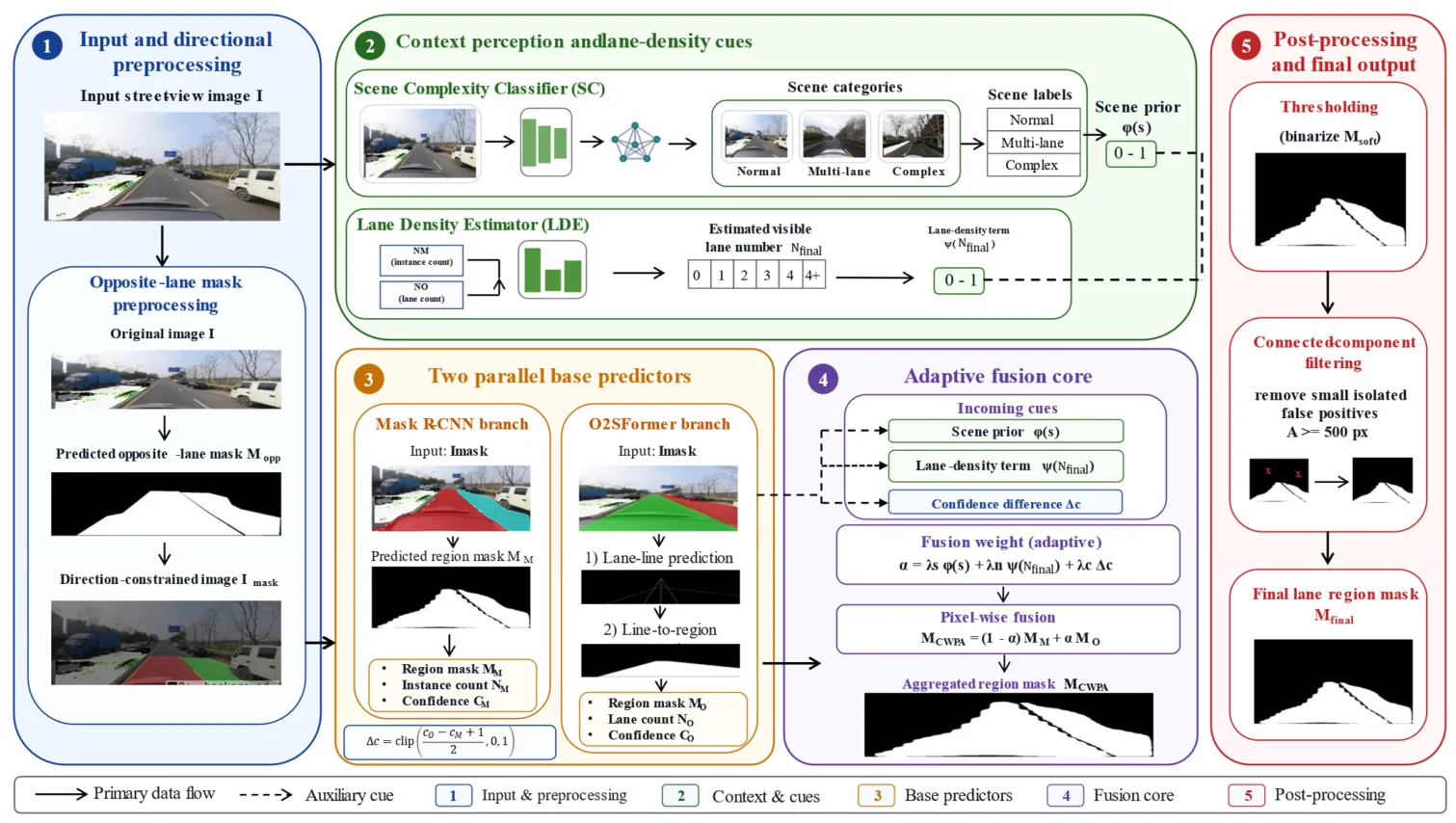
Overview of the ASLIF framework.

ASLIF is a prediction-level structural line–instance fusion framework for direction-consistent lane-area perception from urban street-view images (Figure 1). The conditions for lane-area perception vary widely across street-view images. Under occlusion, low illumination, long-range perspective convergence, or complex road structures, lane lines appear as elongated, sparse, and discontinuous visual targets. In scenes with clear markings and complete lane areas, instance-level segmentation provides more direct spatial support. ASLIF therefore does not rely on one model for all road conditions. Instead, it fuses instance-level area prediction with line-level structural prediction and adaptively adjusts the fusion weights according to scene complexity, lane density, and model confidence.

The ASLIF workflow includes opposite-direction lane-area preprocessing and four fusion-stage components, namely a scene complexity classifier (SC), a lane density estimator (LDE), a confidence-weighted prediction aggregator (CWPA), and a lightweight post-processing module. Given an original street-view image I, SC estimates the road-scene category directly from the raw image. We also use a pretrained opposite-direction lane-area prediction model to generate a binary mask that suppresses opposite-direction lane regions in the original image, producing a direction-constrained image I*_mask_*. The opposite-direction mask is applied only to the two base prediction branches, Mask R-CNN and O2SFormer, and does not change the SC input. LDE infers the visible lane count from the outputs of the two base models. CWPA combines scene category, lane density, and the confidence difference between the two models to determine the adaptive fusion weight and generate a fused mask. Finally, connected-component filtering suppresses small isolated false positives and produces the final lane-area prediction M*_final_*.

### 3.2. Opposite-Direction Lane-Area Mask Preprocessing

**Figure 2:**
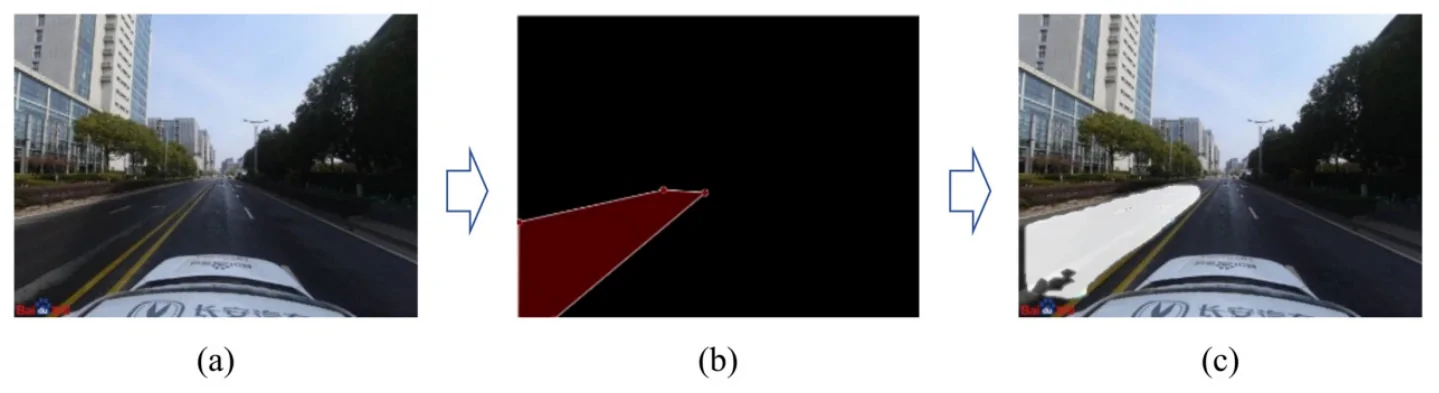
Example of opposite-direction lane-area mask preprocessing. (**a**) Raw street-view image. (**b**) Predicted opposite-direction lane-area mask. (**c**) Direction-constrained image after masking.

This study focuses on extracting motor-vehicle lane areas consistent with the street-view acquisition direction. Some street-view images contain lanes in both directions. Because lanes in both directions share similar pavement textures and lane-marking features, opposite-direction lanes may be incorrectly identified as target lanes, leading to overextended lane-area predictions and overestimated lane counts. To reduce this directional interference, an opposite-direction lane-area mask is introduced before base-model prediction, as illustrated in Figure 2. A separate Mask R-CNN preprocessing model was trained using 500 manually annotated images to localize opposite-direction lane regions. These images were not used for scene-classifier training and were not included in the final lane-area test set. After training, the preprocessing results were manually inspected using a 1300-image evaluation pool that did not overlap with the 500-image preprocessing training set. A preprocessing result was considered successful when opposite-direction lane regions were correctly masked without removing target-direction lane areas. Images without opposite-direction lanes were left unchanged and were also counted as successful cases. The image-level visual assessment yielded a success rate of 99.38%, reflecting the proportion of images satisfying these criteria rather than pixel-level segmentation accuracy. The resulting binary mask is applied before prediction by the two base branches to suppress directional interference in subsequent lane-area prediction and lane-count estimation. During final evaluation, unsuccessful preprocessing outputs were retained without manual correction and passed to the downstream prediction branches.

### 3.3. Scene Complexity Classifier

The scene complexity classifier provides an image-level contextual prior for subsequent adaptive fusion. The three mutually exclusive scene categories are Normal, Multi-lane, and Complex. Normal scenes mainly contain one or two visible lanes under ordinary illumination with clear road structure. Multi-lane scenes contain three or more visible lanes, including parallel multi-lane roads and intersections. Complex scenes include tunnels, nighttime or strong-illumination conditions, severe occlusion, and other visually degraded road environments. To maintain mutual exclusivity, we assign a multi-lane or intersection scene to the Complex category when strong illumination variation, nighttime conditions, or severe occlusion are also present. The scene-classification dataset contains 1000 labeled images, including 400 Normal, 400 Multi-lane, and 200 Complex scenes. Of these, 800 images are used for training and 200 for validation.

An ImageNet-pretrained MobileNetV3-Small [46] is used as the classification backbone. Its classifier is adapted to the three-class task by replacing the final output layer with a linear layer with three outputs. For an input image I, the classifier produces the following scene-probability vector:

(1) $$p=\mathrm{softmax}\;\left(G_{\theta }\left(I\right)\right)={\left[p_{0},p_{1},p_{2}\right]}^{T}$$

where *p*_0_, *p*_1_, and *p*_2_ denote the predicted probabilities of Normal, Multi-lane, and Complex scenes, respectively. The predicted scene category is defined as follows:

(2) $$\hat{s}=\mathrm{argmax}\left(p_{0},p_{1},p_{2}\right)$$

Because the Complex category contains fewer training samples, class-weighted cross-entropy is used during training:

(3) $$L_{\mathrm{sc}}=-\frac{1}{N}\sum_{i=1}^{N}\sum_{k=0}^{2}w_{k}y_{k}^{\left(i\right)}\log p_{k}^{\left(i\right)}.$$

where $y_{k}^{\left(i\right)}$ is the one-hot label of sample *i* for class *k*, $p_{k}^{\left(i\right)}$ is the predicted probability, and *w_k_* is the class weight. The training set contained 320 Normal, 320 Multi-lane, and 160 Complex images. We calculated class weights from the training-set distribution, yielding 0.8333, 0.8333, and 1.6667, respectively. The classifier is trained using AdamW, with the input size, batch size, learning rate, and weight decay summarized in Table 1.

The scene-classification training and validation sets contained no exact image duplicates of the final 300-image lane-area test set. The final test images were not used to train the scene classifier or select its checkpoint. Manual scene labels were also assigned for the 300-image final test set to evaluate the frozen classifier and to analyze how scene-classification errors affect downstream fusion, as reported in Section 4.2. Within CWPA, the predicted scene category is treated as a contextual prior rather than as a direct estimate of branch reliability.

### 3.4. Lane Density Estimator

The lane density estimator derives an image-level visible lane-count estimate from the two base prediction branches. In ASLIF, this estimate serves two purposes. It provides a structural cue for calculating the subsequent CWPA fusion weight and is retained as the final visible lane-count prediction.

The Mask R-CNN branch generates instance-level lane-area candidates. For an input image I, its output prediction set is as follows:

(4) $$P_{M}={\left(m_{i},b_{i},c_{i}\right)}_{i=1}^{N_{M}^{\mathrm{cand}}}.$$

where *m_i_* denotes the probability mask of the i-th instance, *b_i_* is the corresponding bounding box, *c_i_* is the instance confidence score, and $N_{M}^{\mathrm{cand}}$ is the number of candidate instances. Because the raw candidates may include low-confidence false positives, small-area noise, or duplicate instances, valid instances are first selected using the following confidence and area constraints:

Ω*_M_* = {*i*∣*c_i_* ≥ *τ_M_*, *A*(*m_i_*) ≥ *A*_min_}. (5)

When raw predictions were exported, detections with confidence scores of at least 0.05 were retained. For subsequent instance selection, the confidence threshold τ_M_ was set to 0.99, and the minimum instance area A_min_ was set to 500 pixels. The instance area was calculated by thresholding each region-of-interest (ROI) probability mask at 0.10 and counting the foreground pixels. Non-maximum suppression (NMS) in the region proposal network (RPN) and at the final detection stage used IoU thresholds of 0.70 and 0.30, respectively. No additional mask-overlap suppression was applied during downstream instance selection.

The lane-count estimate given by the Mask R-CNN branch is defined as follows:

*N_M_* = |Ω*_M_*| (6)

All valid instance probability masks are then merged into an area-level response map:

(7) $$M_{M}\left(x,y\right)=\underset{i\in \Omega_{M}}{\max}m_{i}\left(x,y\right),$$

When no valid lane instance is retained, the aggregated response map is set to zero at all pixel locations. For standalone evaluation of the Mask R-CNN branch, the aggregated soft response was binarized using τ_eval,M_ = 0.31, without connected-component post-processing:

(8) $$\hat{M_{M}}\left(x,y\right)=1\left[M_{M}\left(x,y\right)\geq \tau_{\mathrm{eval},M}\right]$$

The O2SFormer branch outputs discrete lane-line instances. To obtain a regional representation compatible with the Mask R-CNN response, the line-level predictions are converted into lane-area regions. Let the row-anchor set in the valid road area be as follows:

*Y* = {*y*_1_,*y*_2_,…,*y_K_*}. (9)

Each valid lane-line instance predicted by O2SFormer was fitted with a second-order polynomial and sampled at 5-pixel vertical intervals within its observed range, without extrapolation. Adjacent fitted lines were examined over their longest contiguous common valid run. A candidate pair was retained only when the common vertical support was at least 20 pixels and the pair width at every sampled row remained within [140,639] pixels at the 640 × 480 reference resolution. The sampled left and right boundaries were then joined to form a closed polygon and rasterized as a binary lane-area region. Crossing pairs and candidate regions smaller than 300 pixels were discarded. Missing boundaries were not extrapolated or completed. If fewer than two valid lane boundaries remained after geometric filtering, no line-derived lane region was generated. Because these geometric constraints are defined in pixel units at the 640 × 480 reference resolution, width-related thresholds can be scaled proportionally with image width, while the vertical sampling interval and common-support threshold can be scaled with image height. The minimum region-area threshold can be adjusted according to the corresponding two-dimensional image scale:

(10) $$M_{O}\left(x,y\right)=\underset{\left(j,k\right)\in P}{⋁}1\left[\left(x,y\right)\in R_{\mathrm{jk}}\right]$$

where ℘ denotes the set of valid adjacent lane-line pairs satisfying the geometric constraints, R_jk_ denotes the rasterized region enclosed by the j-th and k-th fitted lane boundaries, and **1**[⋅] is the indicator function. Thus, the line-level predictions from O2SFormer are converted into a binary regional representation that is compatible with the lane-area response produced by Mask R-CNN.

For lane-count estimation, N_M_ denotes the number of valid Mask R-CNN lane instances retained after the confidence and area constraints in Equation (5). For O2SFormer, N_O_ is defined as the number of valid adjacent lane-line pair/region records retained after the common-support, width, crossing, and region-area checks described above. Therefore, N_O_ is not calculated directly from the raw number of detected lane lines.

The final visible-lane-count estimate is defined as follows:

*N*_final_ = max(*N_M_*,*N_O_*) (11)

This max-based aggregation aims to reduce underestimation caused by missed lane instances or missed structural lane regions in either branch. It is not designed to minimize absolute lane-count error for every image. The resulting N_final_ is used both as the lane-density cue in CWPA and as the final visible lane-count estimate reported by ASLIF. Importantly, the lane-count estimate is determined before pixel-level CWPA fusion. Consequently, changing the CWPA weighting strategy or the subsequent connected-component post-processing does not redefine the visible-lane-count estimate.

### 3.5. Confidence-Weighted Prediction Aggregator

CWPA adaptively combines the soft lane-area response generated by Mask R-CNN with the binary regional mask reconstructed from O2SFormer lane lines. Rather than applying a fixed union, intersection, or constant weighting rule to all images, CWPA computes an image-level fusion coefficient α ∈ [0,1] to regulate the relative contribution of the two prediction branches. Here, α controls the contribution of the O2SFormer-derived regional mask, whereas 1 − α controls the contribution of the Mask R-CNN soft response. The coefficient is determined from three image-level cues, namely scene complexity, estimated lane density, and the difference between the raw confidence summaries of the two branches.

The scene-complexity prior $\phi \left(\hat{s}\right)$ is defined as follows:

(12) $$\phi \left(\hat{s}\right)=\left\{\begin{array}{ll} 0.20, & \hat{s}=0,\\ 0.50, & \hat{s}=1,\\ 0.80, & \hat{s}=2. \end{array}\right.$$

(13) $$\psi =\min\left(\frac{N_{\mathrm{final}}}{6},1\right)$$

For the confidence cue, *c_M_* is calculated as the mean confidence score of the retained valid Mask R-CNN lane instances, whereas *c_O_* is calculated as the mean confidence of the retained O2SFormer lane predictions contributing to the line-to-area conversion. If a branch contains no valid prediction, its confidence summary is set to zero. Because the two branches have different prediction forms and their confidence scores are not explicitly calibrated to a common probability scale, the confidence difference is used only as a relative image-level adjustment cue.

(14) $$\Delta c=\mathrm{clip}\left(\frac{c_{O}-c_{M}+1}{2},0,1\right)$$

Combining these three cues, the adaptive weighting coefficient assigned to O2SFormer is computed as follows:

(15) $$\alpha =\lambda_{s}\phi \left(\hat{s}\right)+\lambda_{n}\psi +\lambda_{c}\Delta c,\;\lambda_{s}+\lambda_{n}+\lambda_{c}=1,\;\lambda_{s},\lambda_{n},\lambda_{c}\geq 0.$$

where *λ_s_*, *λ_n_*, and *λ_c_* control the contributions of scene complexity, lane density, and confidence difference, respectively. The scene priors for Normal, Multi-lane, and Complex scenes were set to 0.20, 0.50, and 0.80, respectively, reflecting their ordinal complexity levels. These prior values were fixed during fusion-weight optimization, while the relative contributions of the three cues were determined using the lane-area validation set. The resulting parameters were retained for final test evaluation. Because all three input terms are normalized and the non-negative weights sum to one, *α* lies in [0,1].

CWPA then combines the two regional representations as follows:

*M*_soft_(*x*,*y*) = (1 − *α*)*M_M_*(*x*,*y*) + *αM_O_*(*x*,*y*). (16)

Here, M_M_(x,y) ∈ [0,1] is the continuous Mask R-CNN lane-area response, whereas M_O_(x,y) ∈ {0,1} is the binary regional mask reconstructed from O2SFormer lane lines. Consequently, M_soft_ preserves the graded spatial response of Mask R-CNN while incorporating the structural support provided by the line-derived region. The fusion coefficient is constant within each image and therefore adjusts the relative contribution of the two representations uniformly over the image.

The fused response is subsequently binarized using a global threshold τ:

*M*_raw_(*x*,*y*) = 1[*M*_soft_(*x*,*y*) ≥ τ] (17)

The resulting binary mask is then passed to the connected-component post-processing described in Section 3.6.

### 3.6. Post-Processing and Parameter Optimization

The binary mask M_raw_ obtained after thresholding the CWPA response may still contain small isolated foreground regions caused by local false-positive responses. We therefore apply lightweight connected-component filtering as the final inference-stage post-processing step. Specifically, 8-neighbor connected-component labeling is applied to the foreground mask, and only components satisfying the minimum-area constraint in Equation (18) are retained:

*M*_final_(*x*,*y*) = **1** [∃*k*:*A_k_* ≥ *τ_A_*, (*x*,*y*) ∈ *C_k_*]. (18)

The minimum connected-component area is set to 500 pixels. This operation is intended only to suppress small isolated false positives and does not introduce additional structural reconstruction or boundary completion. The retained mask constitutes the final direction-consistent lane-area prediction *M*_final_. Together with the separately estimated visible-lane count N_final_ from Section 3.4, these form the two outputs of ASLIF. The final regional mask is a merged binary lane-area representation and does not preserve individual lane-instance identities.

Mask R-CNN and O2SFormer are trained separately or initialized from their respective pretrained weights and serve as the two base prediction branches of ASLIF. The scene complexity classifier is the only component that requires separate supervised training within the fusion framework. The CWPA parameters were optimized on 100 images from the lane-area development set. A four-stage search strategy was adopted. First, 27 candidate fusion-weight combinations were evaluated with the binarization threshold fixed at 0.50. Second, the threshold was varied from 0.30 to 0.60 at intervals of 0.025 while retaining the selected weights. Third, 12 alternative formulations of the scene, lane-density, and confidence cues were evaluated. Finally, the fusion weights and binarization threshold were jointly refined by evaluating 27 weight combinations and 5 locally sampled threshold values, resulting in 135 candidate configurations.

Candidate configurations were ranked according to validation micro-F1, with micro-precision and the number of false-positive pixels serving as successive tie-breaking criteria. The highest-ranked configuration in the final search stage yielded fusion weights of (λs, λn, λc) = (1/3, 1/2, 1/6) and a binarization threshold of τ = 0.31, achieving a validation micro-F1 of 0.866715. These parameters were subsequently applied to the final test set without further adjustment:

(19) $$\left(\lambda^{*},\tau^{*}\right)\in \underset{\left(\lambda ,\tau \right)\in G_{D}}{\mathrm{arg max}}F_{1,\mathrm{micro}}\left(M_{\mathrm{final}}\left(\lambda ,\tau \right),G\right)$$

where ***λ*** = (*λ_s_*,*λ_n_*,*λ_c_*) denotes the fusion-weight vector, *τ* is the binarization threshold, and $G_{D}$ represents the 135 candidate configurations evaluated in the final search stage. *M*_final_ denotes the predicted lane-area mask under the corresponding parameter configuration, and *G* represents the ground-truth lane-area masks of the validation set.

### 3.7. Study Area and Dataset

**Figure 3:**
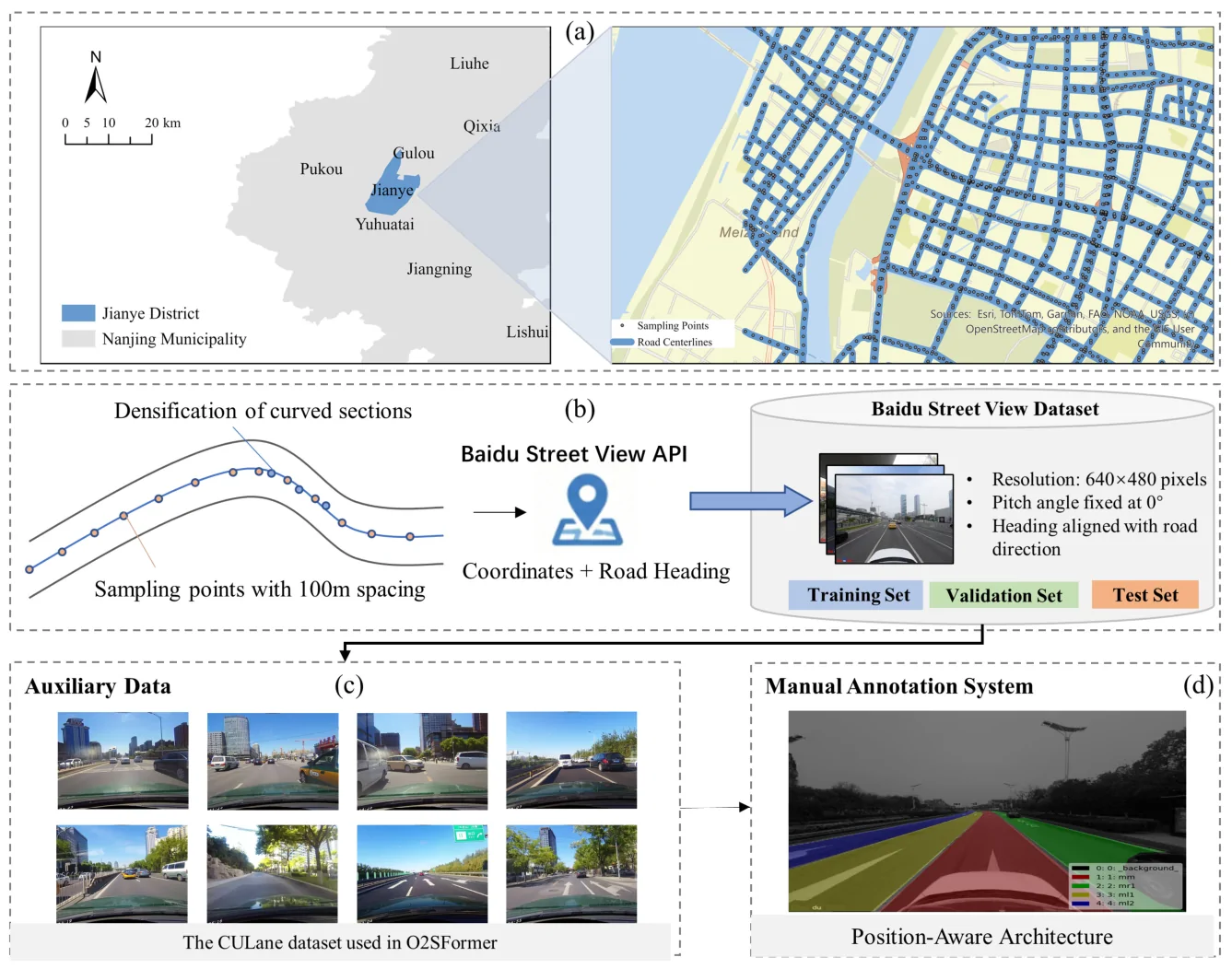
Study area and data. (**a**) Study-area location. (**b**) Data acquisition. (**c**) Auxiliary dataset. (**d**) Manual annotation results.

Jianye District in Nanjing, China, was selected as the study area (Figure 3). The district has a dense road network that includes arterial roads, secondary roads, central medians, side roads, and complex roadside facilities. It also has broad Baidu Street View coverage, providing a broad set of samples for lane-level road-information extraction. OpenStreetMap (OSM) road centerlines were used as the base road-network representation. Pedestrian roads, service roads, and other road types weakly related to motor-vehicle traffic were removed to maintain consistency with the lane-extraction task. Based on the processed centerlines, street-view sampling points were generated at 100 m intervals, with denser sampling along curved segments. The Baidu Street View API was then used to download images in batches using longitude, latitude, and road heading, establishing a correspondence among road centerlines, sampling points, and street-view images. The street-view images were acquired in 2021 at a resolution of 640 × 480 pixels with a pitch angle of 0^∘^, and the horizontal heading was aligned with the road direction. The exact camera field-of-view metadata were not retained in the archived acquisition records. In addition, CULane was used as the pretraining dataset for lane-boundary detection. This dataset covers congestion, shadows, night scenes, strong illumination, missing markings, curves, and intersections. It is therefore suitable as a pretraining basis for street-view lane detection in urban road environments.

### 3.8. Dataset Split and Annotation Protocol

A total of 1400 street-view images were manually annotated for lane-area perception and divided into 800 training images, 300 development images, and 300 final test images. A subset of 100 development images was used for fusion-parameter optimization. The selected parameters were fixed before evaluation on the final test set, which was not used for model training or parameter selection. The dataset was partitioned at the image level, and strict independence between road segments was not established. Consequently, spatial correlations between nearby samples may remain across the subsets.

Manual annotations followed a direction-consistent lane-area definition. Target regions comprised visible motor-vehicle lane areas aligned with the street-view acquisition direction. Opposite-direction lanes, bicycle and other non-motorized lanes, medians, curbs, and unrelated roadside pavement were excluded. Visible public-transport lanes, parking lanes, and multi-use motor-vehicle lanes were included when they were consistent with the acquisition direction. For partially occluded lanes, only the visible lane area was annotated. Occluded portions were not inferred or completed. In the far field, regions with uncertain lane boundaries were excluded rather than extrapolated. At intersections, only lane regions consistent with the current acquisition direction were retained. No separate ignore label was used. All annotations were produced by a single annotator and subsequently re-inspected by that annotator for consistency. Therefore, no independent inter-annotator agreement statistic is reported. The manually verified final test set contained 146 Normal, 114 Multi-lane, and 40 Complex scenes, corresponding to 48.67%, 38.00%, and 13.33% of the 300 test images, respectively.

## 4. Experiments and Results

### 4.1. Implementation Details

Mask R-CNN employed a ResNet-101-FPN architecture initialized with MS-COCO-pretrained weights. It was trained for 100 epochs using Adam with a learning rate of 1 × 10^−5^ and a batch size of 1 on an NVIDIA GeForce RTX 2060 SUPER GPU. During inference, images were resized and padded to a square input, with predictions subsequently restored to the original coordinates. O2SFormer adopted a CULane-pretrained ResNet-34 backbone without additional fine-tuning. The 640 × 480 street-view images were resized to 1640 × 590 pixels, cropped by 270 pixels at the top, and resized to 800 × 320 pixels for inference. Predicted lane coordinates were then mapped back to the original image space for regional reconstruction. The scene complexity classifier employed ImageNet-pretrained MobileNetV3-Small. The original classification layers were retained, except for the final linear layer, which was replaced with a three-class output layer with 1024 input features and 3 outputs. Full images were resized to 256 × 512 pixels without spatial cropping. The classifier was trained for 100 epochs using class-weighted cross-entropy, and the checkpoint with the highest validation macro-F1 was selected for inference. All nine semantic-segmentation baselines used the same 800 manually annotated training images as Mask R-CNN, with an input resolution of 256 × 320 pixels. Mask R-CNN was implemented using TensorFlow 1.13.1 and Keras 2.2.4, while O2SFormer, the scene classifier, and the segmentation baselines used PyTorch 1.13.1. Connected-component analysis was implemented using OpenCV. The main implementation settings are summarized in Table 1.

### 4.2. Scene Complexity Classification Results

The scene complexity classifier provides the scene-level contextual cue used by CWPA. MobileNetV3-Small was adopted as the classification backbone for three-class scene recognition. The classifier was evaluated on the manually verified 300-image final test set. As shown in Table 2, it achieved an overall accuracy of 0.9067 and a macro-F1 of 0.9060. The F1-scores for the Normal, Multi-lane, and Complex categories were 0.9161, 0.8954, and 0.9067, respectively.

**Figure 4:**
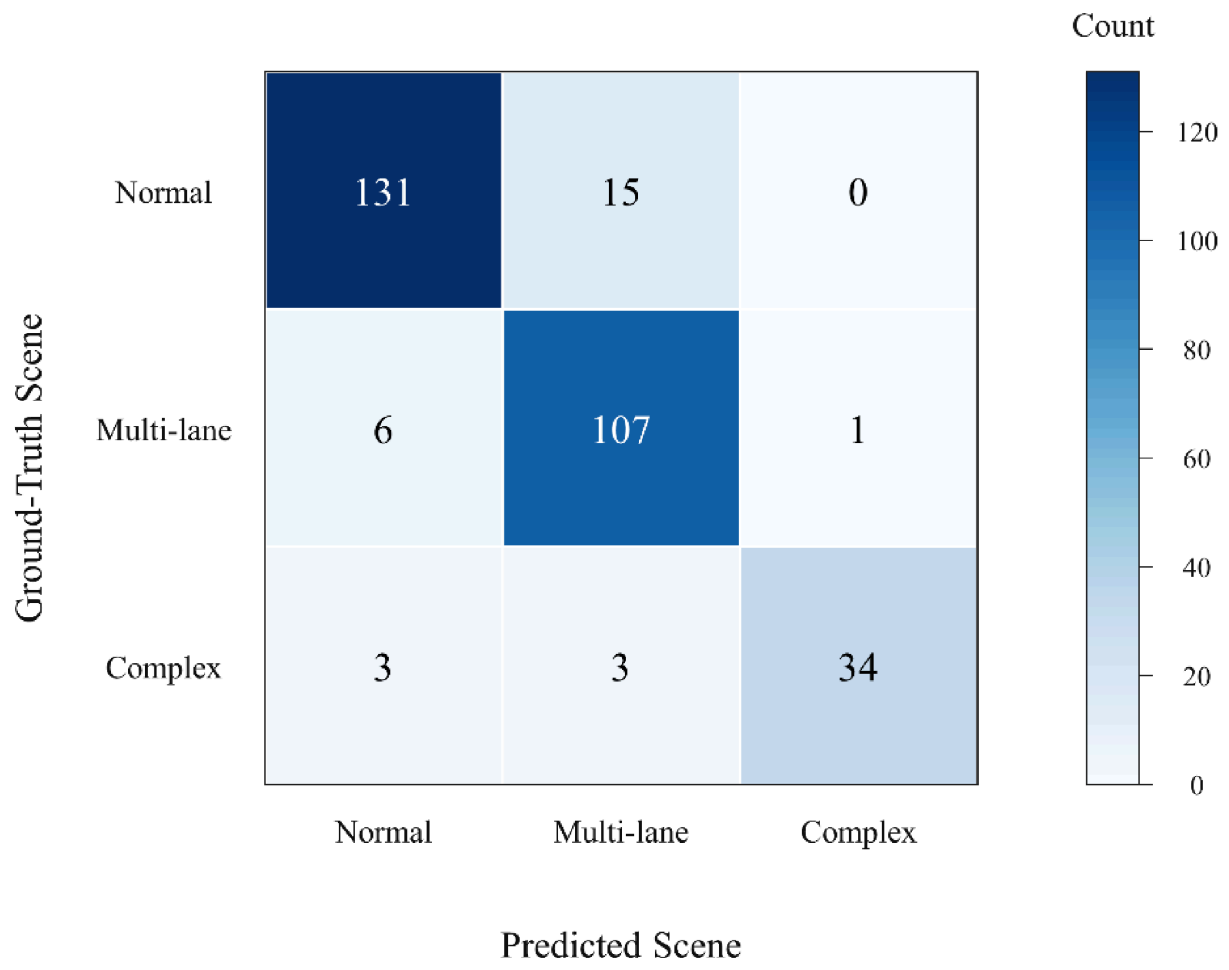
Confusion matrix for the scene complexity classifier on the manually verified final test set of 300 images. Rows represent the ground-truth scene categories, and columns represent the scene predictions used during final ASLIF inference.

As shown in Figure 4, the model correctly classified 272 of the 300 test images. Most errors occurred between the Normal and Multi-lane categories. Specifically, 15 Normal scenes were classified as Multi-lane, whereas 6 Multi-lane scenes were classified as Normal. The Complex category showed high precision (0.9714) but relatively lower recall (0.8500), with six Complex scenes assigned to the other two categories.

To examine how scene-classification errors propagate to the fusion stage, the predicted scene labels of the 28 misclassified images were replaced with their manually verified categories while all other fusion inputs and parameters were kept unchanged. On this subset, micro-F1 changed from 0.8814 to 0.8731 and micro-IoU from 0.7880 to 0.7748. Per-image F1 increased in 8 cases and decreased in 20 cases. These results indicate that correct scene classification does not necessarily yield a fusion coefficient that improves lane-area segmentation performance. Accordingly, CWPA uses the scene category as a contextual prior rather than a direct reliability estimate or branch-selection signal.

### 4.3. Performance of the Base Prediction Branches

**Figure 5:**
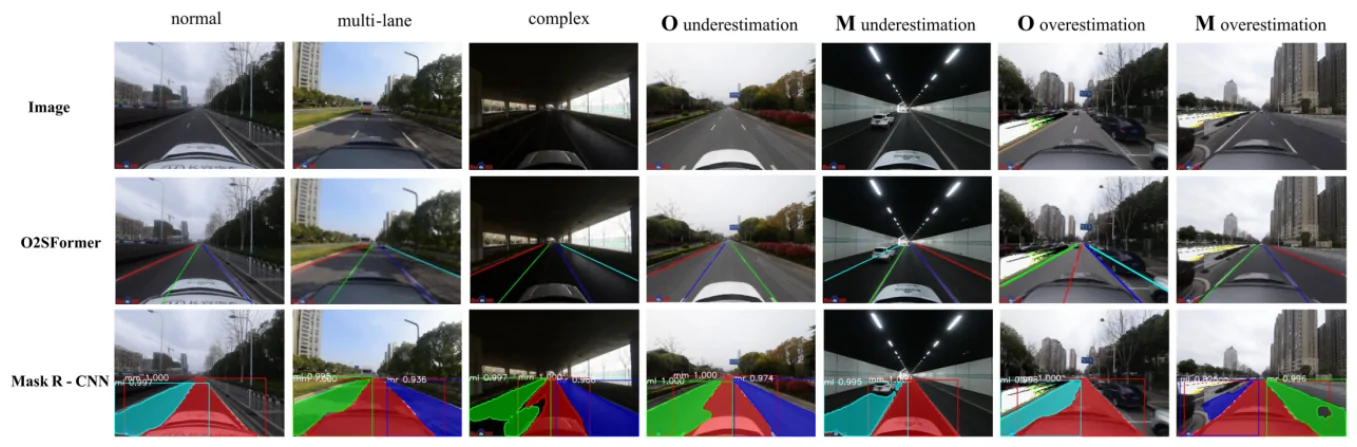
Qualitative results of the two base prediction branches and examples of lane-count estimation errors. O denotes O2SFormer, and M denotes Mask R-CNN. Different colors distinguish individual lane-line or lane-area predictions and do not indicate different semantic classes.

To evaluate the reliability of the lane density estimator, we analyzed the two base prediction branches, Mask R-CNN and O2SFormer. Figure 5 shows their predictions in typical road scenes and in cases with lane-count estimation errors. Overall, both branches extract the main lane structure on ordinary and multi-lane roads, but they show distinct prediction characteristics. O2SFormer outputs lane lines and better preserves the perspective geometry of the road and longitudinal continuity. It can still provide useful line-structure information in complex scenes such as tunnels, shadows, and local occlusion. Mask R-CNN directly generates instance-level lane areas, providing a direct representation of lane extent and instance count. It performs more consistently when markings are clear and lane boundaries are complete. The two branches therefore provide complementary structural and regional information, although their relative performance varies across individual images.

To examine the influence of the line-to-area geometry, we further varied the minimum pair-width threshold on the validation set. Reducing w_min_ from 140 pixels to 120 and 100 pixels decreased the full-system micro-F1 by 0.0061 and 0.0130, respectively. In the middle rows of the image, only 21.3% and 15.5% of the additional pixels introduced by these relaxed thresholds overlapped the reference lane-area masks, indicating that excessive relaxation of the width threshold mainly introduced weakly supported regional responses. Increasing w_min_ to 160 or 180 pixels produced slightly higher fused micro-F1 values but progressively reduced the structural regions generated by O2SFormer. At 180 pixels, the lane-count MAE increased from 0.306 to 0.561. The 140-pixel lower bound was therefore retained to balance regional false-positive suppression, structural coverage, and lane-count preservation.

The remaining geometric parameters showed lower sensitivity within the evaluated ranges. Moderate changes in polynomial degree, vertical sampling interval, and the upper width bound produced relatively small variations in the final result, whereas a stricter common-support requirement reduced the number of generated regions. Varying the minimum region area between 100 and 800 pixels did not change the evaluated predictions. These observations indicate that the minimum pair width is the most influential geometric constraint in this setting.

### 4.4. Adaptive Fusion Mechanism

#### 4.4.1. Adaptive Fusion-Weight Analysis

Table 3 summarizes the image-level fusion coefficient α and its component cues across the predicted scene categories used during final ASLIF inference. The mean α increases from 0.3008 in Normal scenes to 0.4722 in Multi-lane scenes and 0.5421 in Complex scenes. This between-category shift is primarily associated with the scene prior ϕ, while the lane-density and confidence-difference cues provide additional image-specific adjustment.

The standard deviation of α remains non-zero within all three categories, ranging from 0.0317 to 0.0530, indicating that images assigned to the same scene category do not receive identical fusion coefficients. The scene category therefore shifts the fusion coefficient, while the other two cues further adjust the relative contribution of the two prediction branches at the image level.

#### 4.4.2. Ablation Analysis

For the ablation experiments, the standalone Mask R-CNN response was binarized at a threshold of 0.31, while O2SFormer generated binary lane-area masks through line-to-area conversion. Union and intersection fusion were performed directly on these binary branch masks without additional connected-component filtering. The single-cue variants and complete ASLIF combined the continuous Mask R-CNN response with the binary O2SFormer regional mask using a fusion threshold of 0.31. Eight connected components smaller than 500 pixels were removed from these fusion outputs, except for the variant explicitly evaluated without post-processing. All configurations used the same directionally preprocessed input images and ground-truth masks. Thus, comparisons between logical fusion and CWPA reflect differences in both prediction representation and fusion strategy. Table 4 reports lane-area segmentation performance, Table 5 presents the separately estimated visible-lane counts, and Table 6 evaluates opposite-direction preprocessing.

As shown in Table 4, the two base branches exhibit different prediction characteristics. Mask R-CNN provides relatively compact regional responses, whereas the line-derived O2SFormer regions provide greater regional coverage. The logical fusion baselines further reveal the trade-off between these two behaviors. Union fusion increases recall by retaining branch-specific foreground responses, but this also introduces additional false-positive regions. In contrast, intersection fusion produces more conservative predictions and higher precision at the expense of foreground coverage. The single-cue variants demonstrate that scene context, lane density, and branch confidence provide complementary information for image-level fusion. Confidence-only fusion remains close to the Mask R-CNN operating point, whereas the scene-only and lane-density-only variants achieve a more favorable balance between precision and regional coverage. The complete ASLIF formulation maintains high precision while preserving competitive F1 and IoU performance, indicating that combining multiple contextual cues provides more balanced regional predictions than relying on a single fusion cue. The small difference between ASLIF with and without post-processing further indicates that connected-component filtering mainly removes isolated false-positive regions rather than determining the overall prediction structure.

To further assess the differences between the complete fusion formulation and the single-cue variants, we generated 5000 paired image-level bootstrap resamples using identical sampled image indices across configurations. The micro-F1 improvements of full ASLIF over scene-only, lane-density-only, and confidence-only fusion were 0.0117 (95% confidence interval (CI): [0.0088, 0.0145]), 0.0127 (95% CI: [0.0098, 0.0156]), and 0.0371 (95% CI: [0.0341, 0.0401]), respectively. All three intervals were above zero, supporting the observed advantages of the complete formulation over the evaluated single-cue variants. These comparisons do not constitute a leave-one-cue-out analysis and therefore do not establish the independent necessity of each cue.

Table 5 summarizes the visible-lane-count results. O2SFormer achieves the lowest LC-MAE and the highest exact-count accuracy among the three estimators, indicating that its structured line representation strongly supports lane-count inference. The max-based ASLIF estimator yields an LC-MAE of 0.2200 and an exact-count accuracy of 0.8067, indicating slightly lower counting accuracy than O2SFormer alone. Its role is therefore not to optimize MAE directly, but to reduce the risk of underestimating the visible lane count when one branch misses valid lane evidence. Among the 300 test images, the valid-instance counts produced by Mask R-CNN matched the reference lane counts in 204 images, with 44 overestimations and 52 underestimations. These statistics were calculated after applying the instance-selection criteria described in Section 3.4.

The preprocessing ablation in Table 6 evaluates the effect of suppressing opposite-direction lane regions before the two base prediction branches. With preprocessing, precision increases from 0.9086 to 0.9193, micro-F1 from 0.8984 to 0.9035, mean-F1 from 0.8868 to 0.9016, and mean-IoU from 0.8093 to 0.8214, while recall decreases slightly from 0.8884 to 0.8881. LC-MAE decreases from 0.2767 to 0.2200. These results indicate that opposite-direction preprocessing reduces false-positive lane-area responses and improves visible-lane-count estimation on the evaluated test set.

#### 4.4.3. Fusion-Parameter Sensitivity Analysis

**Figure 6:**
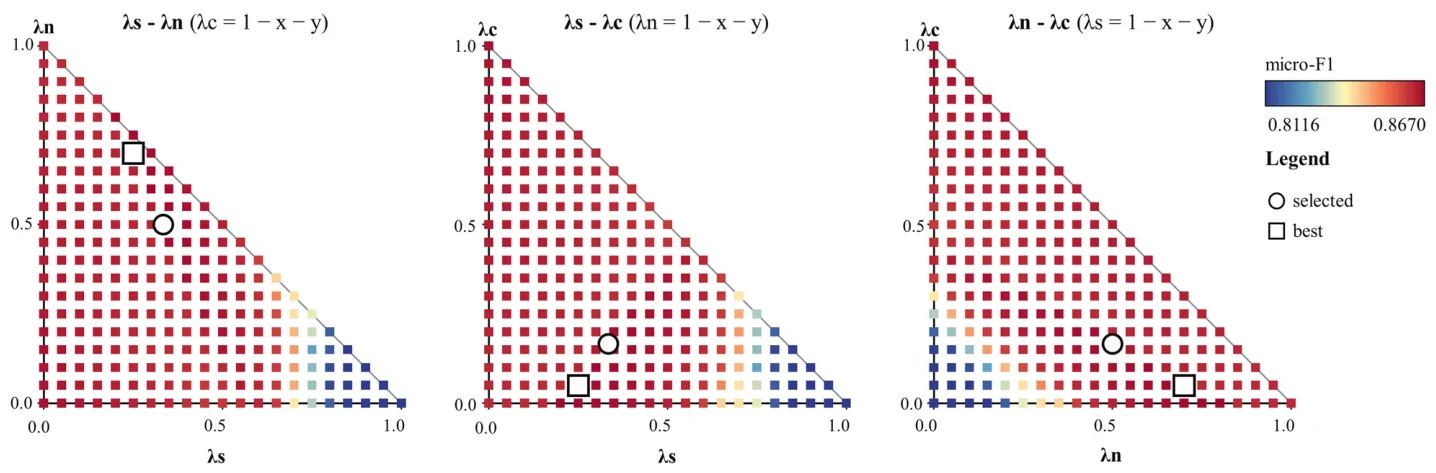
Pairwise sensitivity of the CWPA fusion weights on the validation set at τ = 0.31. Colors indicate micro-F1, while the circle and square denote the selected and best-performing sampled configurations, respectively.

**Figure 7:**
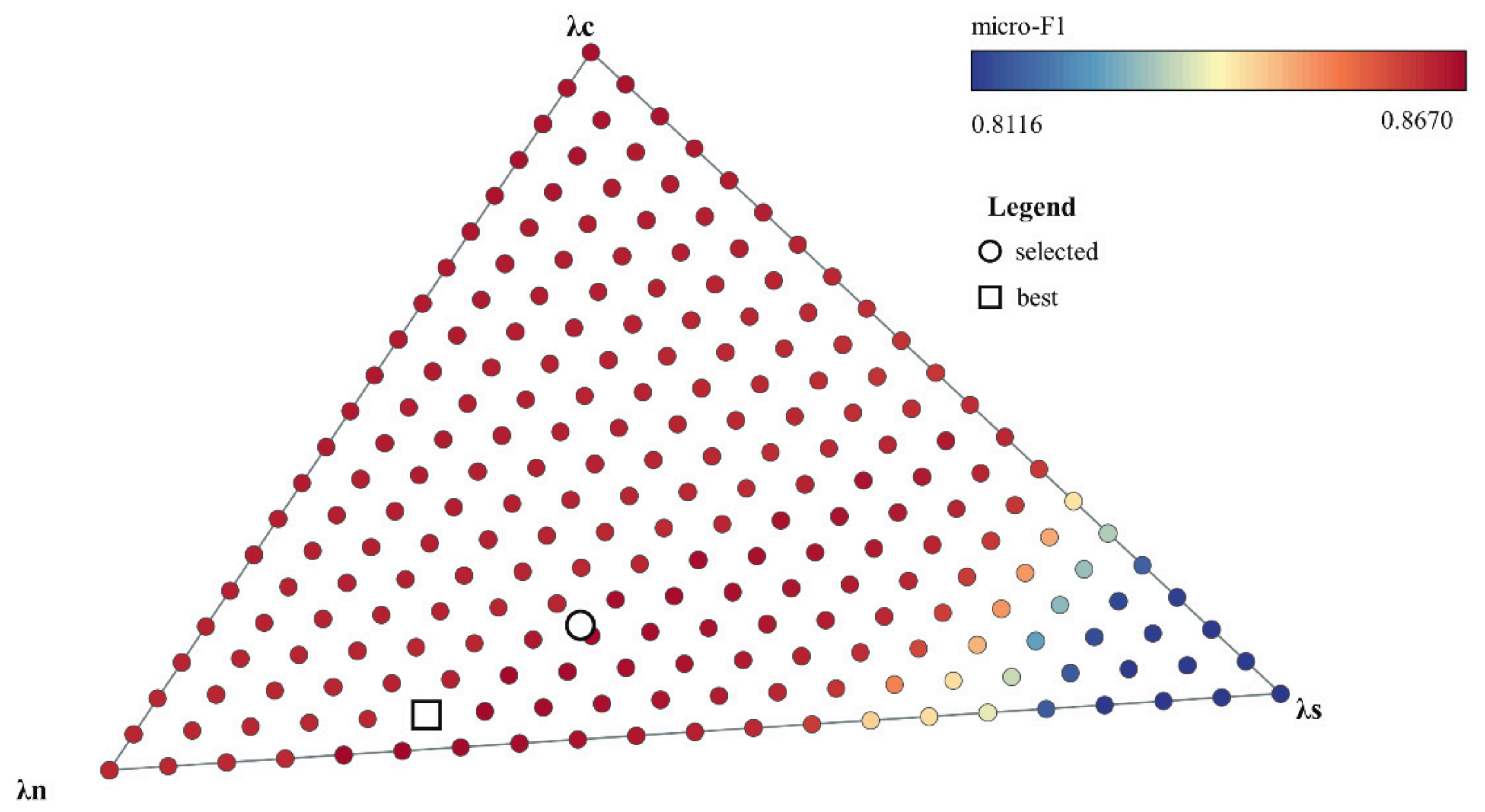
Distribution of micro-F1 across the sampled fusion-weight simplex on the validation set. The circle and square indicate the selected configuration and the highest-performing sampled configuration, respectively.

The sensitivity of CWPA to the fusion weights and binarization threshold was evaluated on the validation set. Figure 6 and Figure 7 illustrate the influence of the relative contributions of the three fusion cues on micro-F1. Higher-performing configurations are concentrated within a region surrounding the selected operating point, indicating that moderate variations in the fusion weights have a limited effect on segmentation performance. The selected configuration achieved a micro-F1 of 0.866715, compared with 0.867044 for the best-performing configuration in the sampled weight space. The selected configuration was obtained from the staged optimization described in Section 3.6, whereas the sensitivity analysis covered a broader set of weight combinations. The two configurations therefore represent the optimum of the formal candidate set and the highest-performing point in the subsequent sensitivity grid, respectively. This small difference suggests that the fusion mechanism is relatively insensitive to moderate changes in the balance among the three cues within this region.

**Figure 8:**
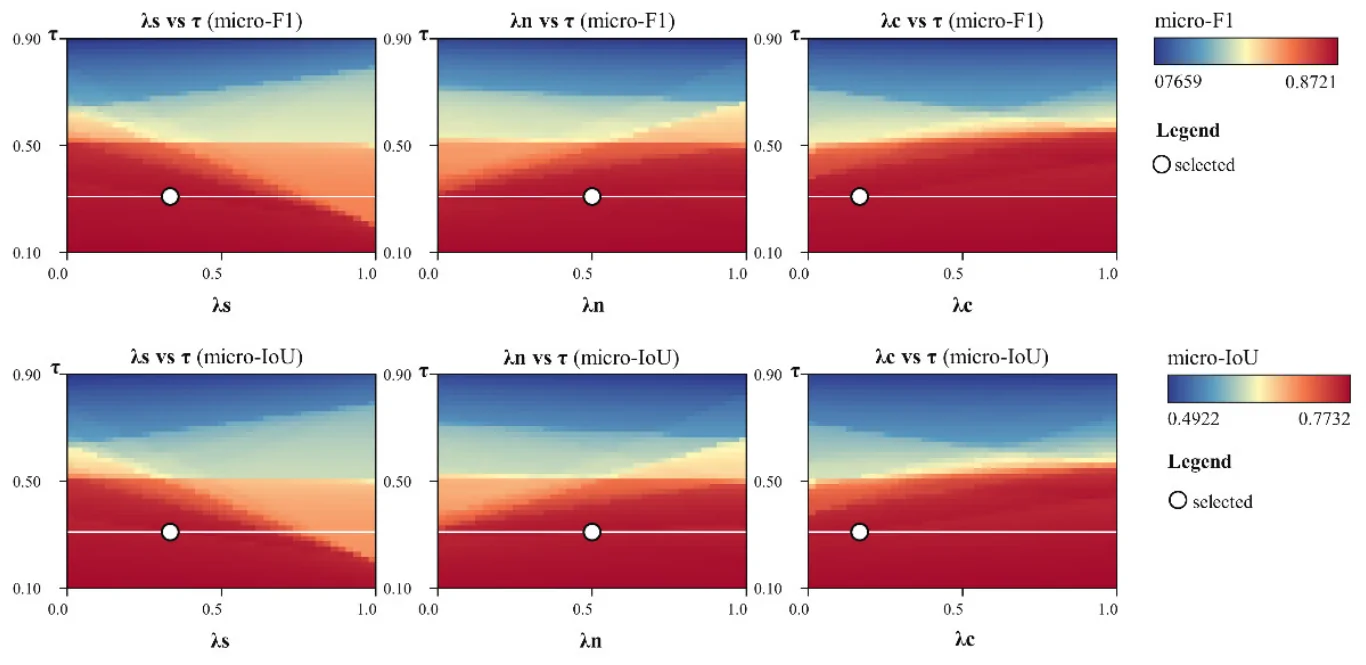
Sensitivity of micro-F1 and micro-IoU to the fusion weights and binarization threshold on the validation set. When one weight was varied, the remaining weights were redistributed proportionally to maintain their sum at one. Colors represent results obtained at discrete sampled configurations.

Figure 8 further illustrates the combined influence of the fusion weights and binarization threshold on micro-F1 and micro-IoU. Compared with moderate variations in the fusion weights, changes in the binarization threshold produce more pronounced performance differences. Lower thresholds retain more weak lane-area responses, whereas higher thresholds increasingly suppress these responses. The highest micro-F1 in the broader sensitivity sweep was 0.872082, obtained at (λs, λn, λc) = (0, 0, 1) and τ = 0.10, outside the candidate set used for formal parameter selection. Overall, the results indicate that the binarization threshold plays a more prominent role in balancing foreground retention and false-positive suppression, while the fusion weights exhibit relatively stable performance around the selected configuration. Although higher validation scores were observed in the broader sensitivity sweep, the configuration established through the staged optimization was retained for final evaluation.

### 4.5. Performance Comparison and Sample-Level Analysis

We compared ASLIF with nine representative semantic-segmentation methods, including DeepLabv3+, ENet, SegNet, ERFNet, SETR, SegFormer, Mask2Former, PIDNet, and SegNeXt. All nine baselines were trained using the same 800 manually annotated lane-area images as the Mask R-CNN branch. During inference, all lane-area prediction methods used images processed by the same opposite-direction lane-masking procedure. Their predictions were evaluated on the same 300-image test set using consistent foreground definitions and evaluation metrics. The comparison therefore reflects differences in segmentation architectures and fusion strategies under a shared directional preprocessing procedure.

Precision, recall, and micro-F1 were calculated from foreground TP, FP, and FN aggregated across the 300 test images. Mean-F1 and mean-IoU were calculated by averaging the corresponding per-image scores over the same test set. All metrics were evaluated against the same foreground lane-area annotations, and none of the test images had an empty ground-truth mask.

To quantify image-level sampling uncertainty, we estimated 95% confidence intervals using 5000 image-level bootstrap resamples. For each method, the final evaluation set was resampled with replacement 5000 times. Precision, recall, micro-F1, mean-F1, and mean-IoU were recalculated for each bootstrap replicate, and the 2.5th and 97.5th percentiles were reported as the corresponding limits of the 95% confidence intervals. Because resampling was performed at the image level, the resulting confidence intervals do not account for potential spatial dependence between nearby images or variability across independent model-training runs.

As shown in Table 7, the evaluated methods exhibit different precision–recall characteristics at their reported operating points. ASLIF achieves the highest precision (0.9193), whereas ENet achieves the highest recall (0.9796), micro-F1 (0.9250), mean-F1 (0.9153), and mean-IoU (0.8545). ASLIF reaches a micro-F1 of 0.9035, a mean-F1 of 0.9016, and a mean-IoU of 0.8214, indicating a more conservative operating point with stronger false-positive suppression but lower foreground coverage. The comparison therefore reflects trade-offs between the reported operating points rather than uniform superiority across all segmentation metrics.

**Figure 9:**
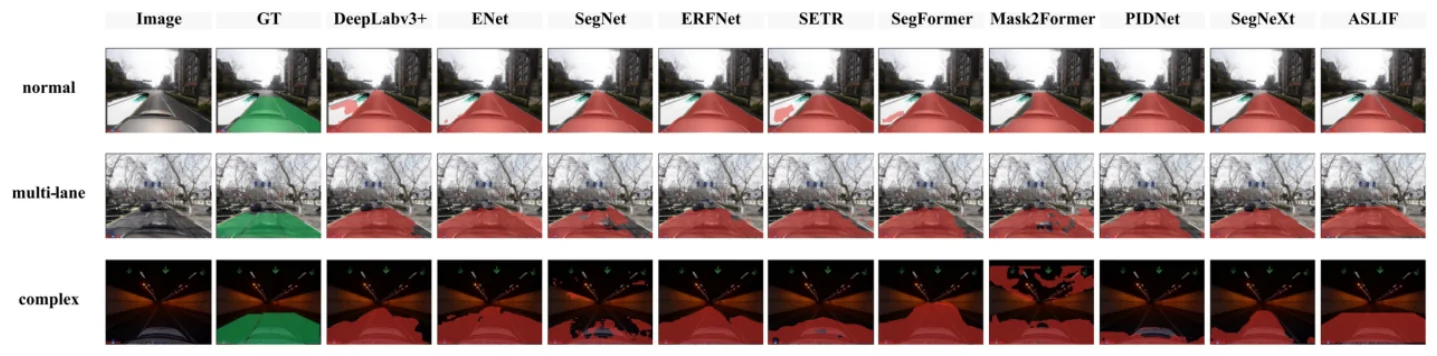
Qualitative comparison of lane-area perception under representative road scenes. ASLIF produced compact target-lane masks and reduced false-positive responses from road edges, medians, and non-motorized lanes compared with several semantic-segmentation baselines. In weak-texture, occluded, or far-range regions, ASLIF occasionally showed under-coverage, consistent with its lower recall and conservative prediction behavior.

In typical road scenes, most methods extracted the main lane area, but some semantic segmentation models extended beyond the lane boundary or included roadside regions. ASLIF predictions more closely matched the ground truth (Figure 9). In multi-lane scenes, vehicle occlusion and complex pavement texture can cause local fragmentation or inclusion of non-lane regions. By combining instance-level area information with line-structure constraints, ASLIF better preserved the main lane shape. In complex low-light scenes, several baselines produced isolated noise or large false-positive regions. ASLIF generated more continuous predictions overall, although some locally blurred regions remained under-covered.

**Figure 10:**
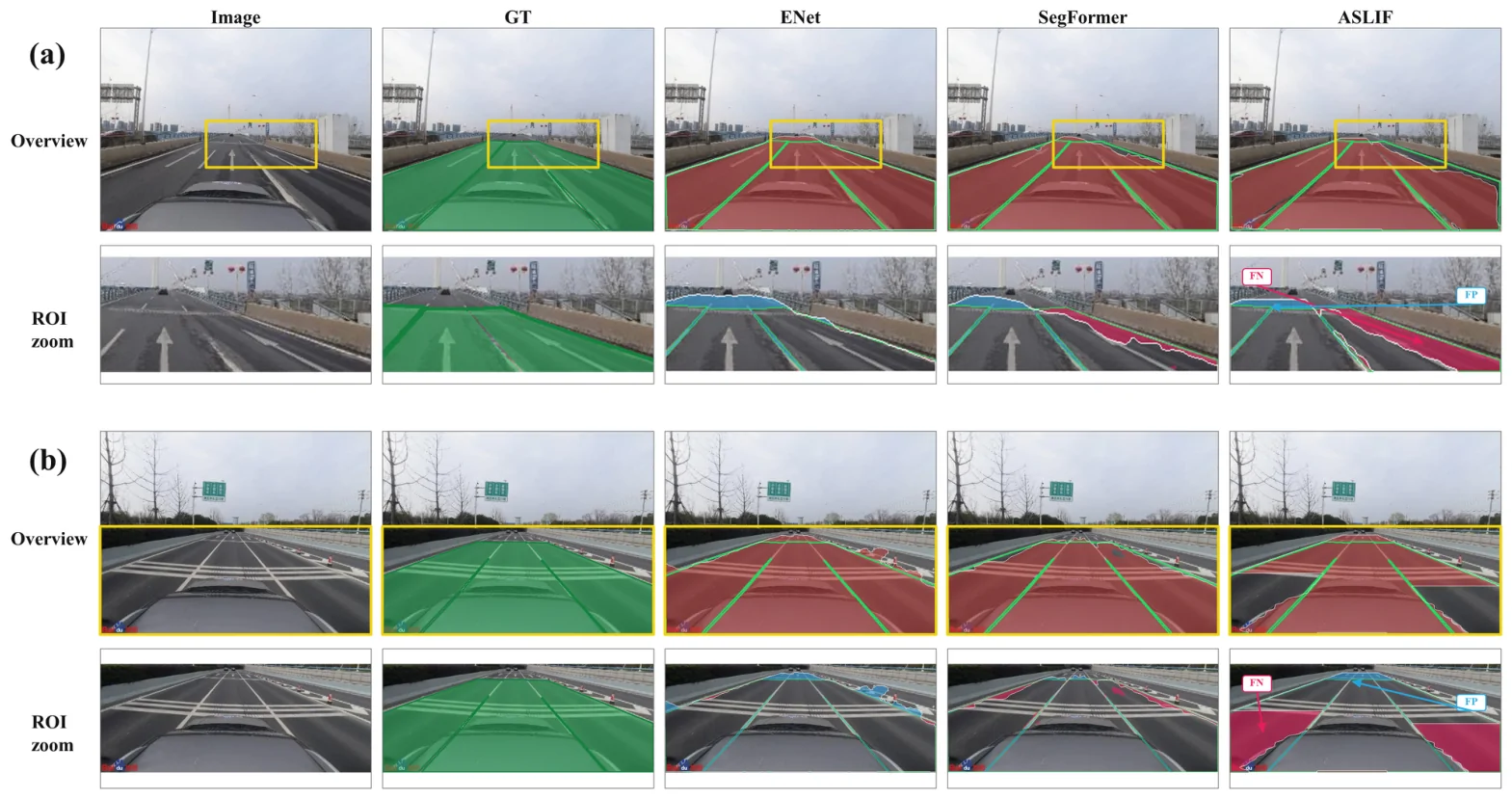
Enlarged qualitative comparison of representative failure cases in challenging road scenes. (**a**) Far-field under-segmentation; (**b**) complex-intersection failure. For each case, the first row shows the overview image and corresponding predictions, and the second row shows the enlarged ROI for detailed comparison. Magenta and cyan denote false-negative (FN) and false-positive (FP) regions, respectively, while green and white contours indicate the ground-truth and predicted boundaries.

To examine residual failure modes more closely, Figure 10 presents enlarged regional comparisons from a far-field under-segmentation case and a complex-intersection failure case.

As shown in Figure 10a, the far-field scene mainly causes under-segmentation near weak or distant lane boundaries, where the predicted lane area is locally incomplete. In Figure 10b, the complex-intersection scene introduces ambiguity in the extent of the lane regions, leading to simultaneous false-negative and false-positive responses in the enlarged region. These enlarged comparisons show that ASLIF retains much of the main lane structure, but local false-negative and false-positive regions remain when lane boundaries and structural evidence are ambiguous.

**Figure 11:**
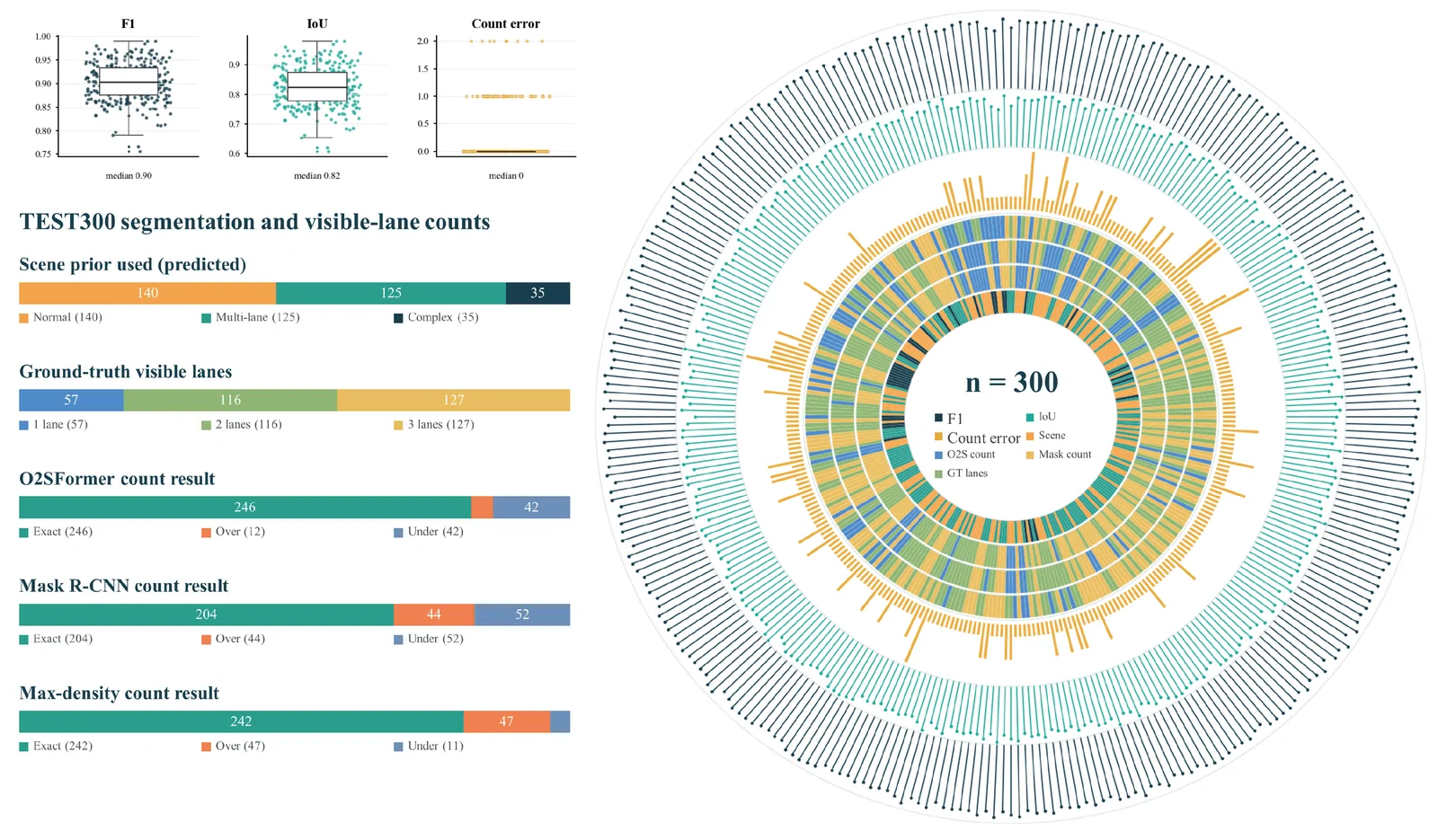
Sample-level performance distribution and lane-count error of ASLIF on the final evaluation set. For most samples, the F1 and IoU values and the lane-count errors are concentrated within narrow ranges, with median F1 and IoU values of 0.90 and 0.82, respectively. Low-performance samples were mainly associated with severe occlusion, perspective convergence, weak markings, or complex intersections. Lane-count error is defined as the absolute difference |N_final_ − N_GT_|.

Figure 11 further presents the sample-level performance distribution on the final test set. The median F1 and IoU were 0.90 and 0.82, respectively, and the median lane-count error was 0. These medians summarize performance on the 300-image study-area test set but do not establish stability across road segments, cities, or acquisition conditions. Overall, ASLIF shows high precision and concentrated distributions of sample-level performance on the study-area test set, supporting the feasibility of combining instance-area prediction with lane-line structural information for lane-area extraction.

Although ASLIF performed consistently in most samples of the study-area test set, a small number of low-performance cases occurred under extreme conditions where both branches lacked reliable visual evidence. These included severe occlusion, long-range perspective convergence, weak or missing markings, strong backlighting, and complex intersections. In such scenes, instance-area boundaries and line-level structures may both degrade. When the fusion module lacks reliable complementary information, it cannot fully recover the true lane area, and predictions may contain local omissions, boundary shifts, or residual false positives. Connected-component filtering can remove small isolated noise, but it cannot correct structural errors produced by the base branches.

## 5. Discussion

### 5.1. Precision-Oriented Performance and Branch Complementarity

ASLIF was proposed to mitigate occlusion, illumination changes, perspective convergence, and complex roadside backgrounds in urban streetscapes for lane-area perception. The method maps Mask R-CNN instance-level area predictions and O2SFormer line-level structural predictions into a common regional-mask space. It dynamically adjusts the relative contributions of the two branches based on scene complexity, lane density, and branch-confidence difference. Previous multitask driving-perception research has shown that lane detection and drivable-area segmentation can be jointly modeled through shared scene representations, providing a basis for the coordinated use of regional information and line-structure information [47]. Among the semantic-segmentation methods compared in Table 7, ASLIF achieved the highest precision (0.92), whereas ENet achieved higher recall, micro-F1, mean-F1, and mean-IoU. This result indicates that ASLIF operates at a more precision-oriented point, with stronger suppression of false-positive lane-area responses but lower foreground coverage. The comparison therefore reflects different precision–recall trade-offs rather than the uniform superiority of one method across all segmentation metrics.

The two base branches complement each other under different road conditions. Mask R-CNN provides reliable instance boundaries and counts when markings are clear and lane areas are complete, but it is vulnerable to roadside structures and complex textures. O2SFormer imposes stronger constraints on longitudinal continuity and lane geometry, making it more robust to weak boundaries, long-range perspective, and complex illumination. However, line-to-area conversion can still be affected by missed lane lines and adjacent-line pairing errors. Prior studies show that global feature aggregation across rows and columns strengthens long-range information transfer for elongated lane structures [48]. Perspective transformation and fine-grained bird’s-eye-view supervision can also alleviate geometric compression and perspective distortion in front-view images [49].

**Figure 12:**
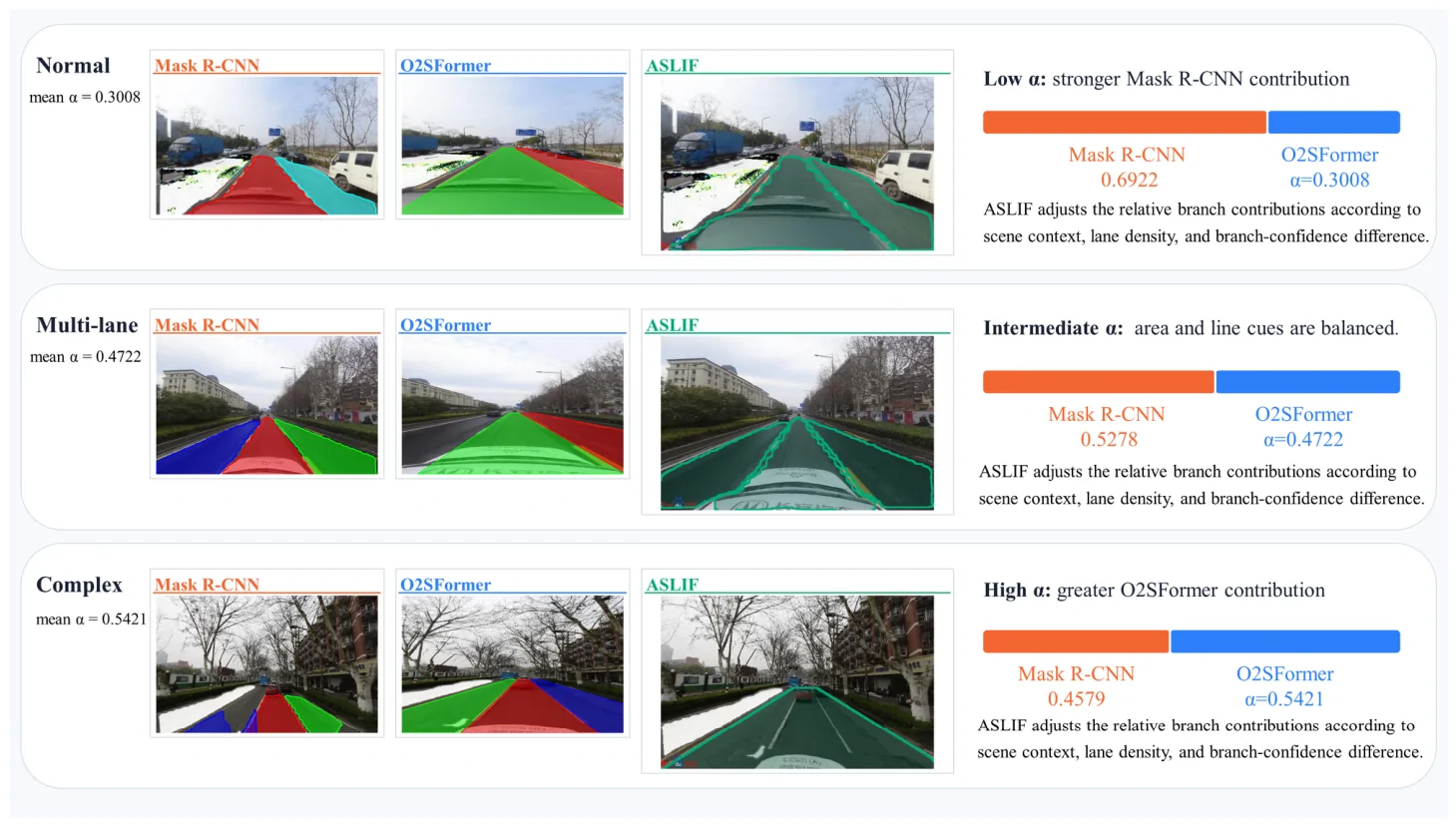
Interpretation of the ASLIF adaptive fusion mechanism under different road scenes. The fusion coefficients and branch-contribution ratios represent averages within each predicted scene category, whereas the images are representative examples.

In addition, learnable anchors and large receptive fields help handle occlusion and complex lane shapes [50]. ASLIF therefore does not simply take a union or intersection of the two predictions. Instead, it uses image-conditioned weighting to resolve disagreements between the two regional representations based on scene suitability. Prior work shows that geometrically consistent matching and confidence learning can improve the relation between prediction score and localization quality [51]. Further evaluation is needed to determine whether the uncalibrated branch scores used by ASLIF provide the same benefit. The combined three-cue formulation achieved higher micro-F1 than the evaluated single-cue variants (Table 4), indicating the benefit of integrating contextual information at the selected operating point. Whether image-conditioned weighting offers further improvements over a validation-optimized fixed-weight fusion strategy remains to be investigated. The scene-dependent weighting behavior of ASLIF is further illustrated in Figure 12.

ASLIF did not achieve the highest performance across all metrics. ENet achieved higher recall, micro-F1, mean-F1, and mean-IoU. The main advantage of ASLIF was higher precision, at the cost of reduced foreground coverage. This operating point may suit road-inventory workflows where false-positive lane regions are more costly than local under-coverage. However, demonstrating practical value requires downstream evaluation, for example, of lane-attribute assignment accuracy or manual correction effort.

### 5.2. Factors Affecting Detection Performance

**Figure 13:**
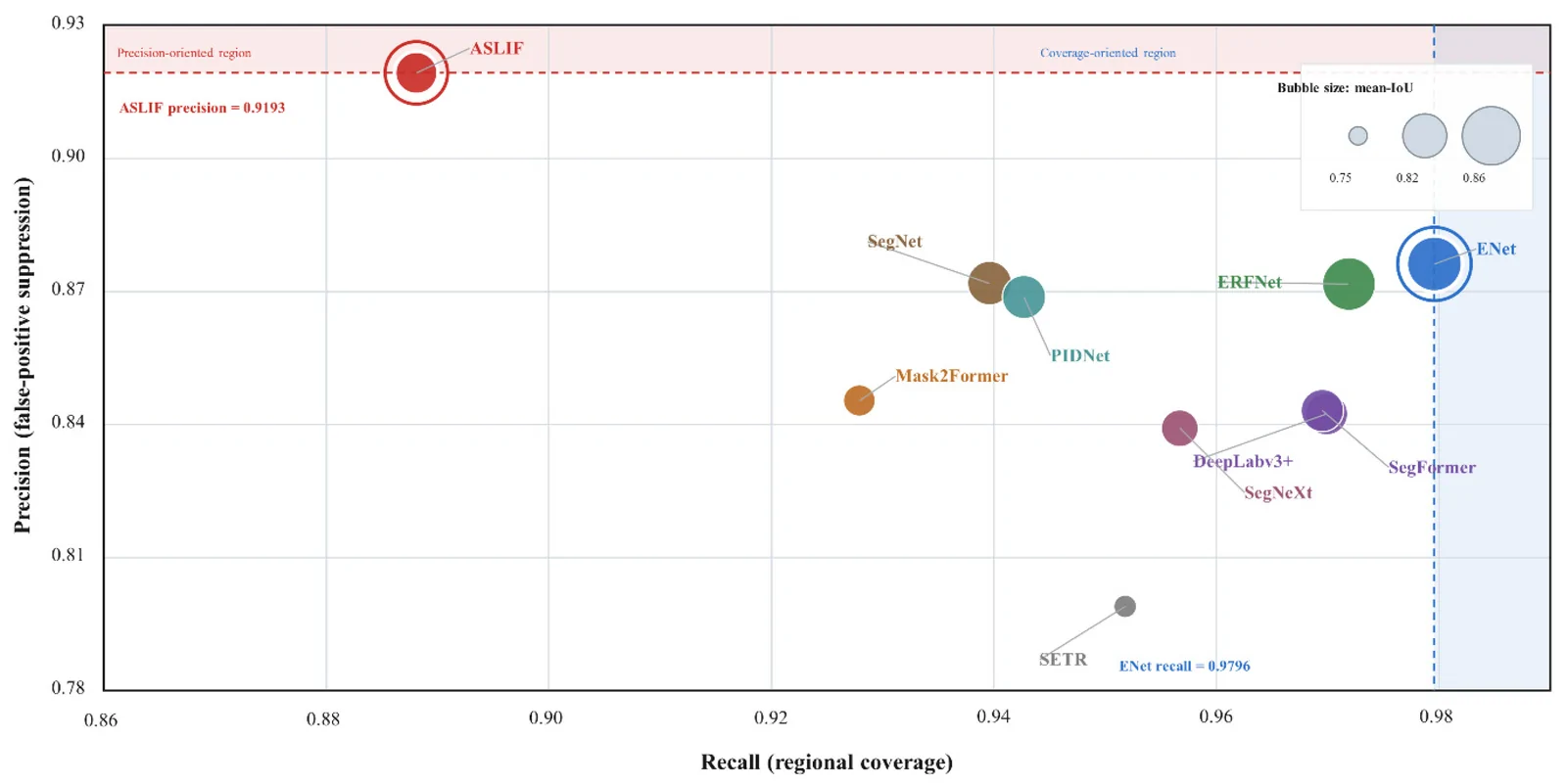
Trade-off between regional coverage and false-positive suppression across lane-area segmentation methods on the final test set. The *x*-axis denotes recall, the *y*-axis denotes precision, and bubble size denotes mean-IoU. The horizontal dashed line marks the precision of ASLIF, and the vertical dashed line marks the recall of ENet.

The observed performance differences may reflect annotation rules, image scale, road environment, and the fusion mechanism. First, delineating lane-area boundaries from lane-line positions and visible road extent is subject to annotation uncertainty, particularly under occlusion. Semantic segmentation research shows that large-context aggregation can blur target boundaries at the cost of lower effective spatial resolution, whereas explicit boundary constraints can mitigate this problem [52]. The ACDC dataset, designed for adverse road conditions such as fog, night, rain, and snow, also includes masks for uncertain regions, indicating that some pixels may lack clear semantics even for human annotators [53]. Long-range perspective convergence, vehicle occlusion, and shadow interference can therefore all cause differences in manually annotated boundaries. Annotation rules that favor area coverage increase recall in semantic segmentation methods. When lane boundaries and instance structure are emphasized, ASLIF has advantages in precision and lane-count representation. The differences among methods therefore reflect different trade-offs between area coverage and boundary reliability. The trade-off between regional coverage and false-positive suppression is further illustrated in Figure 13.

Second, scale variation in street-view images affects the two base branches differently. Near-field lane boundaries and pavement texture are clearer, making it easier for Mask R-CNN to obtain complete instance masks. Far-field lane lines gradually converge into elongated structures. Under these conditions, the line-level geometric constraints of O2SFormer become more advantageous. Research on high-resolution semantic segmentation shows that high-resolution local regions preserve detail, while larger low-resolution regions capture long-range context. These two scales must be coordinated through scale-aware mechanisms [54]. When lane lines are missed, incorrectly ordered, or heavily occluded, line-to-area conversion can still produce local gaps in the reconstructed lane regions. This scale-dependent behavior is an important source of variation in ASLIF performance across samples.

The minimum-width constraint also depends on image position. In the present validation data, no valid candidate pairs were available in the upper image band (rows 0–159), preventing a direct quantitative assessment of the 140-pixel threshold in the extreme upper image region. The row-wise analysis therefore characterizes image-position effects rather than physical distance. In addition, the resolution-scaling rules described in Section 3.4 provide geometric normalization for implementation, but their transferability across different datasets and acquisition conditions remains to be evaluated.

Differences in road environments further affect model generalization. Cities differ in marking styles, marking wear, non-motorized lane design, median facilities, and street-view acquisition conditions. These differences may change the error distributions of the base branches and the scene classifier. Domain-generalization research shows that when test scenes differ from training scenes, both accuracy and uncertainty estimation can degrade substantially in unseen domains, even when source-domain performance is high [55,56]. Cross-region applications therefore require testing the transferability of scene classification and, when necessary, recalibrating the fusion threshold, connected-component area threshold, and weight parameters.

From an algorithmic perspective, ASLIF benefits from the complementary characteristics of the two base branches, while difficult cases may still arise when both branches become unreliable simultaneously. The parameter-sensitivity results show that the proposed fusion remains relatively stable over a broad range of weight settings. In contrast, the binarization threshold mainly controls the balance between foreground retention and false-positive suppression (Figure 8). The image-level fusion coefficient provides a stable scene-conditioned adjustment, but it cannot represent spatial variations in branch reliability within the same image. Future work will therefore investigate improved uncertainty calibration, topology-aware consistency constraints, and robustness under occlusion, blur, extreme illumination, and domain shift.

### 5.3. Limitations and Outlook

ASLIF targets workflows that require direction-consistent lane regions and lane-count information from a single street-view image. Its observed advantage is higher precision, not uniform improvement across segmentation metrics. This characteristic may matter for lane-level road inventory, where false-positive regions can contaminate road-attribute assignment. The applicability of the fused masks to high-definition mapping or urban digital-twin workflows remains to be established through downstream evaluation.

Current evidence is limited to urban street-view images from one district, with image headings broadly aligned with road direction. Transferability to other regions, marking standards, and acquisition systems has not yet been demonstrated. Roads without visible markings, construction zones, adverse weather, severe occlusion, and complex intersections remain failure conditions because both base branches may degrade simultaneously.

There are still some limitations to this study. First, the scene category summarizes multiple visual and structural factors but may not fully capture the continuous variation in road complexity. Second, CWPA applies a single linear fusion coefficient to each image and uses uncalibrated branch-confidence information. This formulation cannot adapt to spatial variations in branch reliability within an image or represent nonlinear interactions between the two branches. Third, the dataset was partitioned at the image level, and the retained records do not permit verification of road-segment-level separation. Images acquired from nearby road locations may exhibit spatial dependence. Consequently, the image-level bootstrap confidence intervals reflect sampling uncertainty under image-level resampling but do not account for road-segment clustering or variability across independent model training runs. Spatially independent evaluation across different road segments and geographic regions is therefore required to further assess the generalizability of ASLIF. Fourth, the multi-stage architecture of ASLIF introduces additional computational overhead compared with a single-network segmentation model. Future work will investigate calibrated or learned reliability weighting, spatially disjoint and cross-city evaluation, computational optimization, and the propagation of errors from the base branches to the fused output.

## 6. Conclusions

This study presents ASLIF, a scene-conditioned prediction-level fusion framework for direction-consistent lane-area perception. ASLIF integrates two complementary types of prediction. Mask R-CNN provides a continuous lane-area response, while O2SFormer produces a binary regional mask from detected lane lines. Their relative contributions are adjusted using scene category, estimated lane density, and branch-confidence difference. On the 300-image held-out study-area test set, ASLIF achieved a precision of 0.92, a micro-F1 of 0.90, a mean-F1 of 0.90, and a mean-IoU of 0.82. It improved the regional-mask metrics over both base branches and achieved the highest precision among the semantic-segmentation methods. The results support a precision-oriented operating point within the study area. Spatially disjoint and external-city evaluation, stronger learned-fusion baselines, and category-specific error analysis are required before broader generalization claims can be made.

## Figures and Tables

**Table 1:** Implementation settings and key parameters of ASLIF.

Module	Parameter	Value	Description
SC	Backbone	MobileNetV3-Small	ImageNet-pretrained
	Scene classes	3	Normal/Multi-lane/Complex
	Input size	256 × 512	Preserves road structure
	Batch size	16	—
	Optimizer	AdamW	—
	Learning rate	5 × 10^−5^	—
	Weight decay	3 × 10^−4^	—
Mask R-CNN	Backbone	ResNet-101-FPN	Instance-level lane-area branch
	Initialization	MS-COCO-pretrained	—
	Output	Instances, boxes, scores	Region and lane-count estimation
	Learning rate	1 × 10^−5^	
	Epochs	100	
O2SFormer	Backbone	ResNet-34	Line-structure branch
	Initialization	CULane-pretrained	—
	Output	Lane point sequences	Converted into regional masks
Line-to-area	Polynomial degree	2	Lane-line fitting
	Vertical sampling step	5 pixels	Within observed support
	Minimum common support	20 pixels	Vertical span
	Pair-width range	140–639 pixels	At 640 × 480 resolution
	Minimum region area	300 pixels	Candidate-region filtering
CWPA	*λ_s_*,*λ_n_*,*λ_c_*	1/3, 1/2, 1/6	Selected by grid search
CWPA	Fusion threshold τ	0.31	Validation-selected
Post-processing	CC threshold	500 pixels	Minimum component area

**Table 2:** Scene-classification performance on the manually verified final test set.

Class	Precision	Recall	F1-Score	Support
Normal	0.9357	0.8973	0.9161	146
Multi-lane	0.8560	0.9386	0.8954	114
Complex	0.9714	0.8500	0.9067	40
Macro avg.	0.9210	0.8953	0.9060	300

Note: The overall classification accuracy was 0.9067.

**Table 3:** Statistics of the adaptive fusion coefficient and its component cues across the predicted scene categories.

Predicted Scene	*n*	Mean *α*	*σ*(*α*)	Mean *ϕ*	Mean *ψ*	Mean Δc
Normal	140	0.3008	0.0530	0.2000	0.3286	0.4191
Multi-lane	125	0.4722	0.0317	0.5000	0.4707	0.4209
Complex	35	0.5421	0.0414	0.8000	0.4048	0.4381

**Table 4:** Lane-area segmentation ablation.

Variant	Precision	Recall	Micro-F1	Mean-F1	Mean-IoU
Mask R-CNN	0.8524	0.8715	0.8618	0.8583	0.7528
O2SFormer	0.8731	0.8927	0.8828	0.8797	0.7862
Union fusion	0.8123	0.9595	0.8798	0.8756	0.7799
Intersection fusion	0.9317	0.8046	0.8635	0.8616	0.7575
Confidence-only fusion	0.8512	0.8821	0.8664	0.8631	0.7601
Scene-only fusion	0.9221	0.8634	0.8918	0.8903	0.8028
Lane-density-only fusion	0.9213	0.8622	0.8908	0.8888	0.8005
ASLIF without post-processing	0.9108	0.8942	0.9024	0.9004	0.8194
ASLIF	0.9193	0.8881	0.9035	0.9016	0.8214

**Table 5:** Visible-lane-count evaluation.

Count Estimator	Definition	LC-MAE	Exact Accuracy
Mask R-CNN	N_M_	0.3867	0.6800
O2SFormer	N_O_	0.2000	0.8200
ASLIF	max(N_M_, N_O_)	0.2200	0.8067

**Table 6:** Opposite-direction preprocessing ablation.

Preprocessing	Precision	Recall	Micro-F1	Mean-F1	Mean-IoU	LC-MAE
Without	0.9086	0.8884	0.8984	0.8868	0.8093	0.2767
With	0.9193	0.8881	0.9035	0.9016	0.8214	0.2200

Note: O2SFormer lane-line outputs were converted into regional masks before lane-area evaluation. LC-MAE denotes the mean absolute difference between the predicted and manually annotated visible lane counts. Lower values indicate better counting accuracy. Exact accuracy denotes the proportion of test images for which the predicted visible lane count exactly matches the reference count.

**Table 7:** Quantitative comparison of lane-area segmentation methods on the 300-image final test set, with 95% image-level bootstrap confidence intervals.

Method	Precision	Recall	Micro-F1	Mean-F1	Mean-IoU
DeepLabv3+	0.8424 [0.83, 0.86]	0.9699 [0.95, 0.98]	0.9017 [0.89, 0.91]	0.8894 [0.88, 0.90]	0.8163 [0.80, 0.83]
ENet	0.8762 [0.86, 0.89]	0.9796 [0.97, 0.99]	0.9250 [0.92, 0.93]	0.9153 [0.90, 0.93]	0.8545 [0.85, 0.87]
SegNet	0.8719 [0.86, 0.89]	0.9396 [0.91, 0.96]	0.9045 [0.89, 0.92]	0.8828 [0.86, 0.90]	0.8208 [0.80, 0.84]
ERFNet	0.8717 [0.86, 0.89]	0.9719 [0.96, 0.98]	0.9191 [0.91, 0.93]	0.9089 [0.90, 0.92]	0.8454 [0.82, 0.85]
SETR	0.7990 [0.78, 0.82]	0.9518 [0.93, 0.97]	0.8687 [0.85, 0.88]	0.8533 [0.84, 0.87]	0.7631 [0.74, 0.78]
SegFormer	0.8432 [0.83, 0.86]	0.9695 [0.96, 0.98]	0.9020 [0.89, 0.91]	0.8916 [0.88, 0.90]	0.8170 [0.80, 0.83]
Mask2Former	0.8454 [0.83, 0.86]	0.9279 [0.90, 0.95]	0.8847 [0.87, 0.90]	0.8609 [0.84, 0.88]	0.7868 [0.76, 0.81]
PIDNet	0.8688 [0.85, 0.88]	0.9427 [0.91, 0.97]	0.9042 [0.89, 0.92]	0.8819 [0.86, 0.90]	0.8198 [0.80, 0.84]
SegNeXt	0.8392 [0.82, 0.86]	0.9567 [0.94, 0.97]	0.8941 [0.88, 0.91]	0.8803 [0.87, 0.89]	0.8017 [0.78, 0.82]
ASLIF (ours)	0.9193 [0.91, 0.93]	0.8881 [0.89, 0.90]	0.9035 [0.90, 0.91]	0.9016 [0.88, 0.90]	0.8214 [0.81, 0.83]

## Data Availability

The data supporting the findings of this study are available from the corresponding author upon reasonable request. Third-party street-view imagery, benchmark data, and road-network data are subject to the access conditions and licensing restrictions of their original providers.
